# Understanding water properties in tumorous murine cells using field-cycling NMR relaxometry

**DOI:** 10.1038/s41598-025-28860-3

**Published:** 2025-12-27

**Authors:** David Faux, Rémi Kogon, Janet Godolphin

**Affiliations:** School of Mathematics and Physics, University of Surrey, Guildford, GU2 7XH UK

**Keywords:** Tumorous tissue, Biomarkers, NMR fast field cycling, Low field NMR, Relaxometry, Biomarkers, Cancer, Oncology

## Abstract

The fixed high magnetic fields (1–7 Tesla) used for magnetic resonance imaging produce resolution suitable for oncology but image contrast is insufficient to determine tumour stage. Fast field cycling nuclear magnetic resonance (FFC NMR) measurements spanning low fields (0.24 mT–0.24 T) provide frequency-dependent longitudinal relaxation rate $R_1(f)$ profiles which allow healthy and pathological tissue to be differentiated. In vivo FFC NMR measurements from healthy and tumorous murine tissue spanning a range of tumour fractions have been interpreted using the 3-Tau model (3TM). Each 3TM fit yields six physically meaningful fit parameters. Statistically significant correlation with tumour fraction is found for two of these parameters, namely the surface-to-volume ratio and bulk water dynamic time constant. These fit parameters therefore act as biomarkers. The sensitivity of the biomarkers to tumour fraction is explained by the net water ingress into tumour cells. The sensitivity of $R_1(f)$ at the lowest field ($f=0.01$ MHz) is explained by changes in the surface-to-volume ratio. $R_1(f=0.01)$ is also a biomarker. The water dynamics at solid surfaces are found to change with tumour fraction probably due to differences in cell wall structure between healthy and pathological tissue. FFC NMR measurements interpreted with the 3TM have the potential to estimate tumour fraction from biopsy samples from humans.

Magnetic resonance imaging (MRI) is a high-resolution imaging technique that exploits nuclear magnetic resonance (NMR) measurements to distinguish between organs in the body. An image is generated by taking a measurement at each voxel forming a two-dimensional slice through the patient, then assigning a greyscale value to each voxel based on the magnitude of the observable. The task of a medical scientist is to select the measurement that produces the best image contrast for a particular application.

MRI operates at a fixed, high magnetic field (1–7 Tesla) and can produce image resolution suitable for oncology. However, at high fields, images do not provide sufficient contrast to determine the tumour stage^1^ prompting researchers to explore low field alternatives such as low field imaging^2,3^, in vivo fast field cycling (FFC) NMR, ex vivo FFC NMR or FFC MRI imaging.^4–7^

The present work uses in vivo FFC NMR to distinguish healthy and pathological tissue. FFC NMR measures the longitudinal (spin-lattice) relaxation time $T_1$, often expressed as a rate $R_1\!= \!T_1^{-1}$, for samples sizes typically a few cubic centimeters. An applied magnetic field aligns the proton spins of the water molecules along the field direction before a radio frequency pulse rotates the spins away from equilibrium. The longitudinal relaxation time $T_1$ is a measure of the time taken for the spins to return to equilibrium. The relaxation process requires energy exchange with the surroundings and $T_1$ is determined by the efficiency of the energy exchange process. The energy exchange efficiency is, in turn, determined by the strength of the interactions with nearby spins and the relative dynamics of the pairs of spins. The longitudinal relaxation rate $R_1(f)\!= \!T_1^{-1}(f)$ is measured for magnetic fields of strength *B* in Teslas in the low field regime, typically 0.24 mT to 0.24 T, corresponding to proton Larmor frequency *f* in the range 0.01–10 MHz where $f=\gamma B / 2 \pi$ and $\gamma = 42.58$ MHz/T is the proton gyromagnetic ratio. The $R_1(f)$ dispersion profile captures information about the dynamical environment of water in hydrated porous material with applications to numerous material classes.^8–10^ Example applications in soft matter include proteins^11–15^, macromolecules and biofluids^4–7,16–24^, food technology^25–28^ and hydrogel.^29^

The challenges to developing FFC NMR for assessing tumour stage are twofold. Firstly, FFC NMR experiments normally demand samples of order $1\,\text {cm}^3$ and, secondly, rapid field switching and measurements are required to generate $R_1(f)$. For in vivo applications, the first challenge limits resolution and the second leads to long measurement times. Nonetheless, compromise solutions offering single scan FFC NMR measurements either in vivo or from biopsy material have the potential to provide an assessment of tumour progression.^4–7^

In 2018, Ruggiero and co-workers^19^ conducted in vivo FFC NMR measurements on healthy and tumorous murine tissue samples and found, at low fields (<0.2 T), $R_1(f)$ decreased with increasing tumour fraction. Ruggiero and co-workers did not fit a relaxation model to the $R_1(f)$ profiles. Instead, the sensitivity of the low field relaxation rate to tumour fraction was analysed in terms of intracellular and extracellular exchange rates characterized by time constants $\tau _\textrm{in}$ and $\tau _\textrm{ex}$ respectively. The authors adopted a two-site exchange (2SX) model to construct FFC NMR dispersion (NMRD) profiles from two relaxation rates $R_\textrm{1in}$ and $R_\textrm{1ex}$ determined from fits to magnetization recovery profiles. The NMRD profiles were then constructed from a 2SX model chosen by comparing $\tau _\textrm{in}^{-1} + \tau _\textrm{ex}^{-1}$ to $|R_\textrm{1ex} - R_\textrm{1in}|$. The authors concluded that the water exchange rate across the plasmalemmal membrane differentiated between healthy and tumour cells and that the dynamic properties of the intracellular water dominated the relaxation rate at the lowest magnetic fields.^19^ No attempt was made to generate a relaxation rate model and to fit to the NMRD profiles. The role of the two water exchange time constants $\tau _\textrm{in}$ and $\tau _\textrm{ex}$ was to act alongside the volume fraction as weighting factors for the two NMRD profiles $R_\textrm{1ex}$ and $R_\textrm{1in}$.

The 2SX model of Ruggiero and co-workers was applied in subsequent FFC NMR studies in which relaxation rate enhancement was achieved using gadolinium-based contrast agents. For example, tumour cell cultures were investigated for cells grown under normo and hypoxia conditions.^20^ The significant decrease of the lifetime of water molecules in cell cytoplasm, $\tau _\textrm{in}$, signalled $\tau _\textrm{in}$ as a biomarker for the metabolic change. The same team later conducted in vivo FFC NMR relaxometry experiments to compare healthy tissue and tumour stroma in vivo^21^ to yield dynamic constants for water exchange across cell membranes and relaxation properties for the intra and extracellular components. In 2021, Ruggiero and co-workers used fixed field and low field NMR to find that $\tau _\textrm{in}$ acts as a biomarker for the therapeutic effect of doxorubicin as a treatment for breast cancer. The in-vivo FFC NMR experiments revealed a large difference in $R_1$ values between the intra and extracellular components at low magnetic field strengths.^22^

The NMRD profiles $R_1(f)$ capture information about the nano-scale structure and dynamics of water and the FFC NMR experiment is sensitive to dynamical timescales captured by the range of frequencies explored by the experiment. The lowest frequency, 0.01 MHz, is most sensitive to water dynamics characterized by a time constant of about 100 $\mu$s. Alcicek and co-workers^30^ undertook relaxation rate measurements at zero to ultra-low fields to a maximum frequency of 250 Hz, allowing the investigation of slow (bio)chemical processes occurring on a timescale from milliseconds to seconds. Their maximum frequency (250 Hz) is 50 times smaller than the typical minimum frequency used in FFC NMR experiments. The transmembrane water exchange time constants found through the work of Ruggiero and co-workers^19–22^ for $\tau _\textrm{in}$ and $\tau _\textrm{ex}$ lie in the range 0.02-1.3 s and are therefore examples of biological processes that are too long to impact NMRD profiles. For this reason $\tau _\textrm{in}$ and $\tau _\textrm{ex}$ cannot be used directly as part of a relaxation model to generate model NMRD profiles. However, a two-site water exchange model (2SWEM) *has* been used to produce model NMRD profiles. Guevara and co-workers reported FFC NMR experiments on haemoglobin^23^ and human serum albumin (HSA) solutions.^24^ The model operates on the same principles as the 2SX model in that the number of “sites” refers to the number of dynamical time constants. For the 2SWEM, Lorentzian expressions for $R_1(f)$ relates each time constant to a relaxation rate which are combined to provide a model that can be fit to NMRD profiles. Guevara *et al* reported that the 2SWEM failed to fit the generated NMRD profiles for either the healthy haemoglobin A or sickle cell haemoglobin S solutions and a three-site model (3)SWEM) was preferred.^23^ For the HSA solutions by contrast, both the 2SWEM and 3SWEM returned similar good-quality fits to experimental NMRD profiles.^24^ The two (or three) time constants that provided the optimum fits fell in the range $10^{-8}-10^{-7}$s. However, as the models are not grounded in relaxation theory, the physical meaning of the time constants required educated guesswork. The workers made the reasonable assumption that the motion of proteins about axes of rotation were responsible.

In this study, the 32 NMRD profiles generated by the Ruggiero and co-workers’ murine tissue project^19^ are fitted using the 3-Tau Model (3TM) developed by Faux and co-workers.^31–34^ The 3TM is chosen for four reasons: 3TM delivers a wide range of physically-meaningful fit parameters, has proven transferable to soft material^29,35^, is the only model available capable of capturing both ${\text {Fe}}^{3+}$–^1^H and $\vphantom{0}^1$H–^1^H spin pair interactions, and 3TM fitting software is available open source.^36,37^

The present study uses the 3TM to generate a set of physically-meaningful fit parameters for each of the 32 NMRD profiles from the Ruggiero and co-workers’ project^19^. The objectives are to determine which of the physical quantities show a statistically-significant change with tumour fraction and to explain the sensitivity of the low field relaxation rate to tumour fraction.

## Methods

### Experimental methods

Ruggiero and co-workers^19^ conducted in vivo FFC NMR measurements on healthy and tumorous murine tissue samples. Mouse mammary adenocarcinoma cells were sourced from three different suppliers and labelled here as TSA, 4T1 and FARN. The three cell lines were chosen because they display different aggressiveness and metastatic potential with FARN<TSA<4T1. The cell cultures were injected into the muscle of one of the hind limbs of the mouse to obtain tumour grafts suitable for in vivo studies. The tumour volume was determined from $T_1$-weighted MRI images of the anaesthetized mouse using ITK-SNAP software. The second hind limb acted as a control. The mouse was then placed in the FFC NMR equipment (STELAR, Mede, PV, Italy) with a specially adapted wide bore magnet large enough to host a mouse. The measurements were taken using a bespoke transmitter/receiver solenoid placed around the mouse leg which protruded through a hole outside the primary housing. The full details of the experimental procedures, measurements and analysis are contained in reference^19^ and its Supplementary Material together with the EU and Italian ethical review information.

### The 3-Tau model

**Fig. 1:**
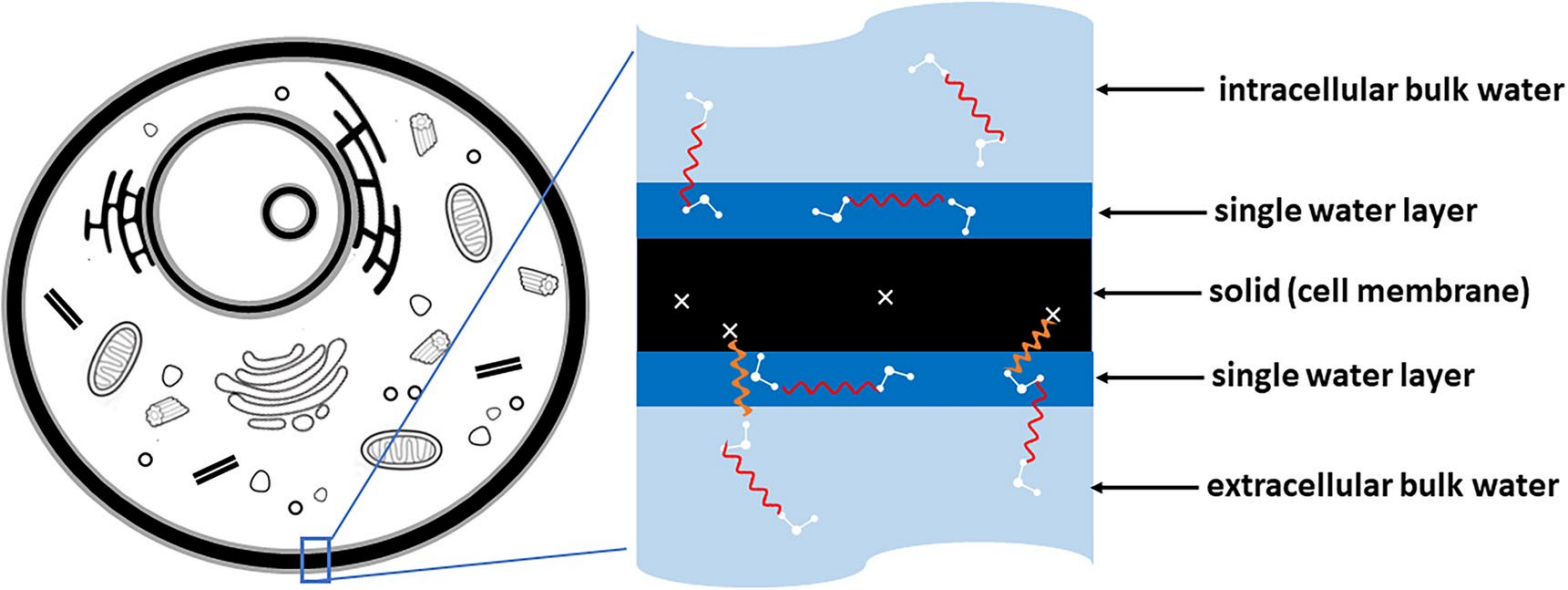
An animal cell (left) shows “solid” components in black. The 3-Tau Model (right) defines a “solid” as any material with a single layer of water of thickness $\delta \!=\!2.7$ Å at its surface including single large molecules, lipids, DNA and proteins. As an example, a section of the cell membrane is shown with specific interactions between spins included in the 3-Tau Model illustrated by wavy lines. Electronic spins due to fixed rare paramagnetic ions are shown as white crosses.

The principles of the 3TM are illustrated in Fig. 1. There are two water environments; a layer of water at the “solid” surfaces of assumed thickness $\delta \!=0.27$ nm, and the bulk water of unspecified thickness. The “solid” relates to any substance with a layer of water, including macromolecules such as proteins and lipids. The dynamical time constants are listed and described in Table 1. Time constants $\tau _\ell$ and $\tau _d$ describe the dynamics of the surface water. The bulk water represents all water not in the surface layer and has a dynamical time constant $\tau _b$.

**Table 1:** Parameters provided by fits to $R_1(f)$ NMRD curves by the 3-Tau Model.

Physical quantity	Symbol	Comments
Surface water dynamical time constant	$\tau _\ell$	The 2-dimensional diffusion coefficient for surface water is $\delta ^2/4 \tau _\ell$. Typically 0.01–10 $\mu$s
Desorption time constant	$\tau _d$	Surface water is assumed to desorb to the bulk with time dependence $\exp (-t/\tau _d)$
Bulk water dynamical time constant	$\tau _b$	The 3-dimensional diffusion coefficient for bulk water is $\delta ^2/6 \tau _b$. $\tau _b \approx 5$ ps for *pure* water
Surface-to-volume ratio	*x*	Equal to $S \delta /V$ where *S* and *V* are the total solid surface area and volume respectively
Surface ^1^H spin density	$N_{\ell }$	Typically 20–40 $\text {spins/nm}^3$ which is approximately 0.3–0.6 of the spin density of water
Paramagnetic ion spin density	$N_\sigma$	The most common paramagnetic ion in mammals is iron

Here $\delta = 0.27$ is a distance between the oxygen atoms of neighbouring water molecules.

All proton-proton interactions included in the 3TM are illustrated in Fig. 1. The ^1^H spins of the surface water interact with other surface spins and bulk ^1^H spins. The bulk water spins interact with other bulk water spins and with surface spins. In addition, the “solid” may contain paramagnetic electronic spins that interact with both the surface and bulk ^1^H spins. Other fit parameters included in the 3TM are listed and described in Table 1.

The HM and HH models describe the two classes of spin pair interaction that contribute to the measured relaxation rate $R_1(f)$. The HM model captures the relaxation rate contribution due to interactions between the electronic spins of rare paramagnetic ions dispersed in the “solid” with surface and bulk protons. The relevant equation is

1 $$\begin{aligned} R_1^{\text {HM}}= & x R_1^{\sigma \ell } + (1-x)R_1^{\sigma b} \end{aligned}$$

where $\sigma$ labels paramagnetic ions, $\ell$ refers to the protons in a surface layer of water, *b* labels the bulk water protons, and *x* is the surface-to-volume ratio.

The HH model captures interactions between pairs of protons as illustrated in Fig. 1. The contributions between proton spin pairs in the different environments combine as follows,

2 $$\begin{aligned} R_1^{\text {HH}}= & x (R_1^{\ell \ell } + R_1^{\ell b}) + (1-x)(R_1^{b b} + R_1^{b \ell }) \end{aligned}$$

where the first and second terms on the right-hand side are the overall contributions to the relaxation rate due to relaxation of the surface and bulk proton spins respectively.

Details of the mathematics and computations of the individual contributing relaxation rate components of Eqs. (1) and (2) may be found in reference^32^. The relaxation rates are pre-calculated for discrete values of the time constants (16 logarithmically spaced values per decade) and put in datafiles. The datafiles are read by the 3TM fitting software producing model relaxation rate components sensitive to the model parameters listed in Table 1. A 3TM fit yields six physically meaningful fit parameters for each NMRD profile.

### The fitting procedure

The NMRD profiles from all 32 experimental datasets were analysed using the 3TM model.^32^ Following Ruggiero and co-workers^19^, the samples are sub-divided into three sets labelled 4T1 (11 datasets), FARN (11 datasets) and TSA (10 datasets) dependent on the original cell culture supplier. All datasets were provided by Stelar s.r.l who supplied the adapted FFC NMR relaxometer for the original experiments.^19^

There is a well-known experimental issue associated with the switch between pulse sequence protocols at a frequency typically 6–10 MHz. The measurements of $R_1(f)$ occasionally exhibit a discontinuity at the switching frequency. Not only is this a common feature of most of the 32 datasets here, it is also a feature of our analyses of other soft materials.^29,35^ In the present analysis the data beyond 8 MHz are excluded from the fitting. Further, in some datasets but not all, a weak broad peak is apparent at 2 MHz almost certainly due to the quadrupolar interaction between ^1^H and ^14^N. ^17^For consistency, two data points about 2 MHz are also excluded from the fitting process for all 32 datasets.

Satisfactory fits could not be obtained with the HH model alone. However, once the HM model was included, excellent fits were obtained to all 32 datasets. The editable MATLAB files are supplied as Additional Files together with a spreadsheet containing input datasets, 3TM fit outputs, analysis and plots.

### Statistical methods

The 3TM fit parameters listed in Table 1 that describe the NMRD profile for each of the 32 samples represent meaningful physical quantities. The objective is to establish which of these physical quantities show a statistically-significant change with tumour fraction. The fit values of $R_1(f)$ at the lowest frequency of 0.01 MHz is also analysed.

The following statistical analysis was conducted. The datasets from each of the three suppliers, 4T1, FARN and TSA, are labelled as $i=1,2$ and 3 respectively. Variables are labelled $y_{i,j}$ where *y* refers to a physical quantity listed in Table 1 or $R_1(f)$ at $f\!= 0.01$ MHz. The subscripts *i*, *j* therefore refer to the $j^{\text {t}h}$ dataset from the $i^{\text {t}h}$ supplier. Two models are then considered and referred to as “parallel” and “coincident”. Both models assume that the variable *y* changes linearly with tumour fraction *c* and that the gradient is independent of culture supplier. The “parallel” model allows the intercept at $c\!=\!0$ (healthy cells) to be different for each culture supplier. The “coincident” model sets the same intercept in each case. The models are described mathematically by

3 $$\begin{aligned} \text{ parallel } \text{ model } y_{i,j}= & \alpha _i + \beta c_{i,j} + \epsilon _{i,j} \end{aligned}$$

4 $$\begin{aligned} \text{ coincident } \text{ model } y_{i,j}= & \alpha + \beta c_{i,j} + \epsilon _{i,j} \end{aligned}$$

where $c_{i,j}$ is the tumour fraction for the $j^{\text {t}h}$ observation from the $i^{\text {t}h}$ supplier expressed as a percentage, and $\epsilon _{i,j}$ is a “noise” term equal to the difference between the model and observation. The $\beta$ represents a common gradient. Both models are tested for each fit parameter *y*.

All datasets used for the statistical analysis are supplied as Additional Files and a summary of the output is provided in Table 2. The statistical significance of the gradient $\beta$ is determined by the *p*-value with $p<0.05$ considered significant. The adjusted coefficient of variation $R^2$ indicates the proportion of the variability in *y* that can be accounted for by tumour fraction *c*. A biomarker is indicated by a small *p*-value combined with a large $R^2$. The statistical analysis was performed with RStudio version 4.3.0.

**Table 2:** Statistical summary.

Physical quantity	Model	Gradient	*p*-value	Adjusted	% change for
				$R^2$	$0\!\le \!c\!\le \! 100$%
$R_1^*$	Coincident	−0.121	$4.34\times 10^{-12}$	0.7959	−41%
$\tau _\ell$	None suitable	$-2.515\times 10^{-4}$	$4.67 \times 10^{-6}$	0.0694	−32%
$\tau _d$	Coincident	$-3.347 \times 10^{-4}$	$1.59\times 10^{-4}$	0.3627	−24 %
$\tau _b$	Parallel	−0.238	$1.33 \times 10^{-8}$	0.7568	−57 %
*x*	Parallel	$-3.076\times 10^{-5}$	$1.32\times 10^{-3}$	0.7202	−23 %
$N_\ell$	None suitable	$2.079\times 10^{-2}$	0.471	0.3295	10 %
$N_\sigma$	None suitable	$-1.899\times 10^{-3}$	0.377	0.6471	−7 %

$R_1^*$ is the relaxation rate at the lowest frequency of 0.01 MHz. A *p*-value less than 0.05 signals a significant gradient. The adjusted coefficient of determination $R^2$ indicates the proportion of the variability with the dataset that can be accounted for by tumour fraction. Where the model is listed as “none suitable”, the parallel model is used for the gradient. The final column provides the fractional change over the full range of tumour fraction.

## Results

### General observations

**Fig. 2:**
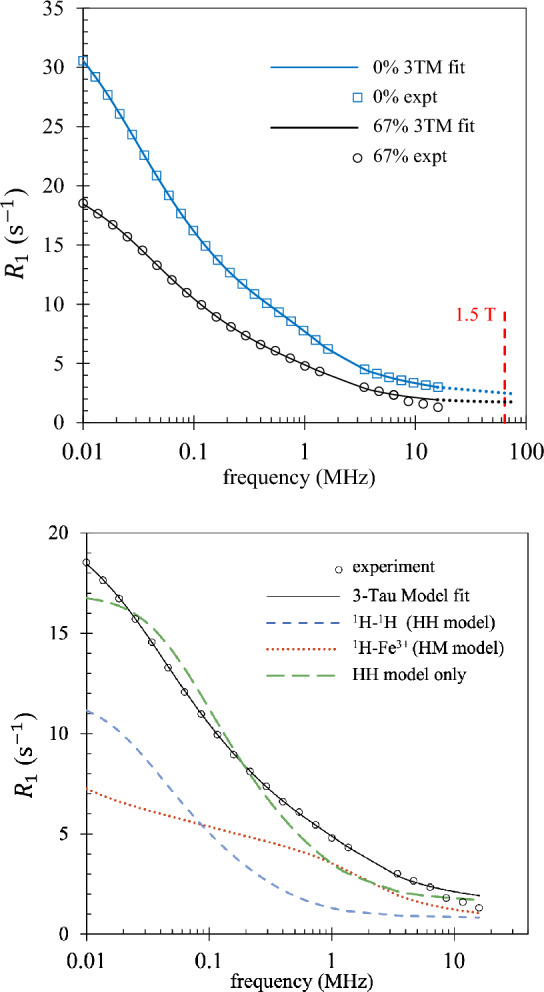
(Top) Example of 3TM fits (solid lines) to experimental data for the same mouse at $c=0$% (healthy) and at tumour fraction $c=67$%. The convergence at a field of 1.5 T is illustrated. (Bottom) The $c=67$% results are repeated with the two model contributions which sum to produce the 3TM fit. The long-dash line is the best fit obtained with the HH model alone.

Figure 2 (top) compares NMRD profiles from healthy tissue and 67% tumorous tissue for the same mouse. The profiles are easily distinguishable at low fields but converge at high fields with a typical MRI magnetic field of 1.5 T shown for reference. Pathological and healthy tissue are therefore more readily distinguished at low fields compared to high fields.

The HM model describes the relaxation rate due to interactions between fixed paramagnetic ions in the “solid” and mobile protons, and is proportional to the paramagnetic spin density $N_\sigma$. The two most significant electronic spin species found in mammals are $\text {Fe}^{3+}$ and $\text {Mn}^{2+}$ ^38^each with spin $S\!=\!5/2$. However, the concentration of $\text {Mn}^{2+}$ is too low by more than two orders of magnitude to account for the observed signal. Therefore, the source of the paramagnetic ion component to the $R_1(f)$ dispersion is assumed to be $\text {Fe}^{3+}$.

The separate HM and HH model contributions for tumour fraction $c\,=\,67\%$ are indicated in Fig. 2 (bottom). Both HM and HH models contribute to $R_1(f)$ at all frequencies. At the higher frequencies, NMRD profiles are sensitive to the fast proton dynamics associated with bulk water. The HM profiles are therefore dominated by interactions between bulk water and paramagnetic ions, and the HH profiles by interactions between bulk water and surface water. By contrast, the NMRD profiles at low fields are sensitive to slow proton dynamics. At low fields the HM model is therefore dominated by the interactions of paramagnetic ions with slow-moving surface water and the HH model is dominated by the interactions between pairs of surface water protons. The relaxation rate at low fields is also sensitive to the surface-to-volume ratio *x*. The best fit obtained using the HH model alone is also included in Fig. 2 (bottom) to demonstrate that interactions of water with paramagnetic ions must be included to achieve satisfactory fits to the experimental NMRD profiles.

### Statistical interpretation

**Fig. 3:**
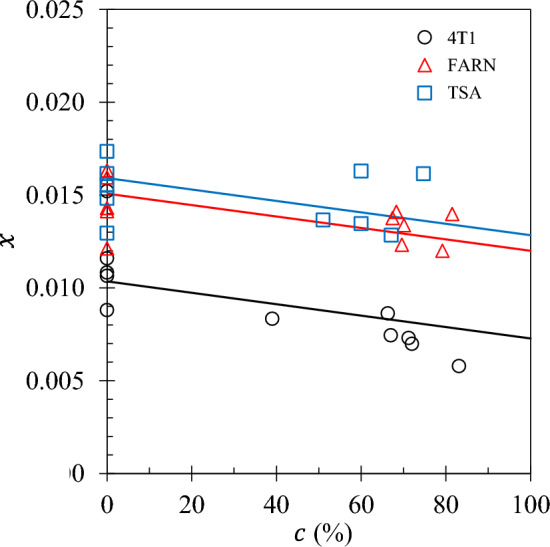
The surface-to-volume ratio *x* obtained from the 3TM fits is plotted as a function of tumour fraction *c* for sets 4T1 ( ), FARN ( ) and TSA ( ). The lines represent output from the parallel model (see text).

The parallel model is the best model describing the surface-to-volume ratio *x* with $p=0.0013$ indicating a highly significant gradient. See Fig. 3. The adjusted $R^2$ for the parallel model suggests 72% of the variation in *x* can be accounted for by the tumour fraction *c*. The surface-to-volume ratio *x* can therefore be considered a biomarker.

**Fig. 4:**
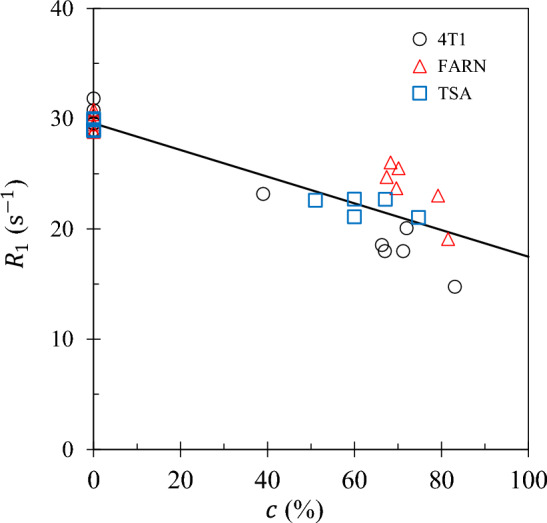
The value of $R_1(f=0.01$ MHz) is plotted as a function of tumour fraction *c* for sets 4T1 ( ), FARN ( ) and TSA ( ). The coincident model was the best model (see text and Eq. (2). The solid line presents the single same gradient and coincident intercept for each culture supplier.

The largest changes in $R_1$ are observed at the lowest field ($f\!=\!0.01$ MHz) and are presented in Fig. 4 as a function of *c* for all datasets. A coincident model with gradient of −0.121 is obtained with very high significance meaning that both the intercept at 0% tumour fraction ($c=0$) and the gradient are independent of the original cell culture supplier. The coincidence of the three models of $R_1$ and the 41% decline of $R_1$ across the range of tumour fraction from 0% to 100% makes $R_1(f=0.01$ MHz) an excellent biomarker.

An explanation for the low field sensitivity of the relaxation rate can be provided as follows. The NMRD profiles at low frequencies are most sensitive to the slow dynamics of the surface water where $R_1(f)$ is approximately proportional to the surface-to-volume ratio *x*. See Eqs. (1) and (2). At the lowest frequency of 0.01 MHz, $R_1(f)$ decreases with increasing tumour fraction *c* primarily because *x* decreases with increasing *c* (Fig. 3).

**Fig. 5:**
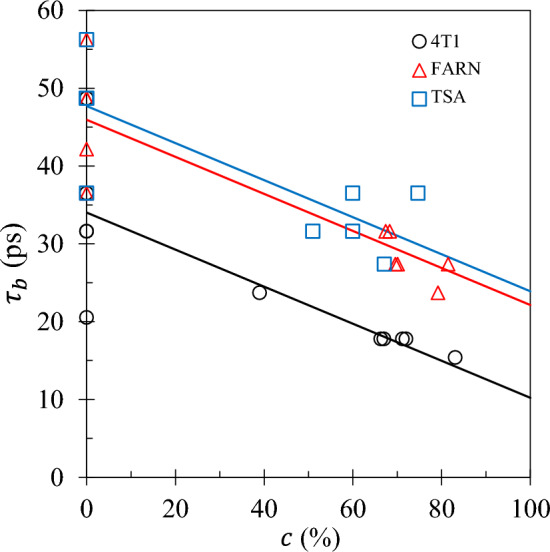
The bulk water dynamical time constant $\tau _b$ obtained from the 3TM fits is plotted as a function of tumour fraction *c* for each set 4T1 ( ), FARN ( ) and TSA ( ). The lines represent output from the parallel model (see text).

The bulk water dynamical time constant $\tau _b$ presents highly significant gradients with *p*-values of 1.3$\times 10^{-8}$ and 4.7$\times 10^{-6}$ for the parallel and coincident models respectively. See Fig. 5. Both models are appropriate with the parallel model presenting the largest $R^2$ with 76% of the variation in $\tau _b$ accounted for by changes in tumour fraction. $\tau _b$ presents the highest sensitivity to tumour fraction with a 57 % reduction across the full range of *c*. The bulk water dynamical time constant $\tau _b$ can therefore be considered a biomarker.

The time constants associated with surface water dynamics, $\tau _\ell$ and $\tau _d$, present statistically significant gradients for both parallel and coincident models. However, the very small $R^2$ for $\tau _\ell$ shows that variation in $\tau _\ell$ cannot reasonably be accounted for by changes in *c*. Neither model is considered useful. For $\tau _d$, both models indicate that 36% of the variation in $\tau _d$ can be accounted for by tumour fraction.

The surface water density $N_\ell$ does not yield gradients that are significantly different from zero. See Table 2. $N_\ell$ cannot therefore be considered a biomarker.

**Fig. 6:**
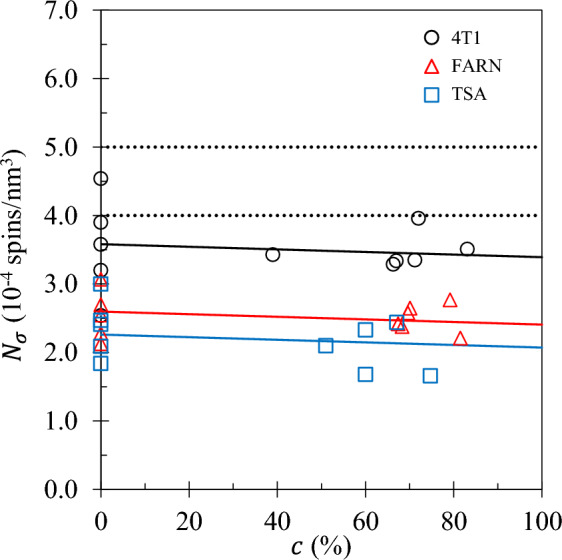
The paramagnetic iron density $N_\sigma$ is plotted as a function of tumour fraction for each set 4T1 ( ), FARN ( ) and TSA ( ). The solid lines represent linear fits and the dotted lines indicate the minimum and maximum range of the iron concentration in an average human.

**Fig. 7:**
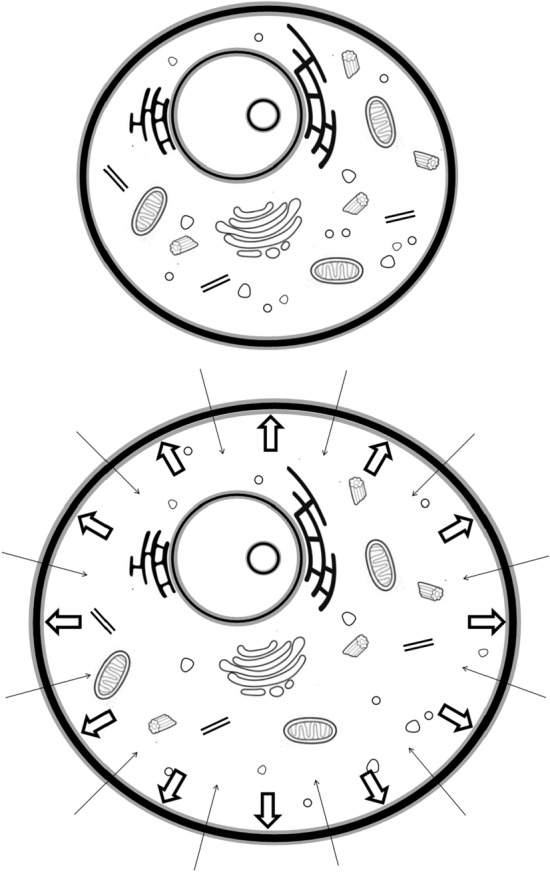
The revised model consistent with the variation of fit 3TM parameters with tumour fraction is shown. A tumour cell (bottom) contains the same components as the healthy cell (top) but increased water content.

The fit values of the paramagnetic ion spin density, $N_\sigma$, are presented in Fig. 6. Neither coincident nor parallel model is useful, with gradient for neither model being significantly different from zero. See Table 2. The paramagnetic ion density is independent of tumour fraction *c* and cannot therefore be considered a biomarker.

### A proposed model

The simplest transformation from healthy cell to tumour cell consistent with the decline of both $\tau _b$ and *x* is presented in Fig. 7. The ingress of water from the extracellular region into the tumour cells leads to a physically larger cell. The surface-to-volume ratio *x* of an idealised spherical cell membrane decreases because the cell volume increases as $r^3$ and the surface area as $r^2$ for a sphere of radius *r*. Cell surface irregularities make the relationship more complex. The ingress of water also causes a decrease of $\tau _b$, as seen in Fig. 5, because the density of intracellular solids and molecules acting as obstacles to diffusion is smaller, and because water exchange between fast-moving bulk and slow-moving water bonded to the solid components becomes less likely. An increase in diffusion coefficient translates to a decrease of $\tau _b$ as observed.

## Discussion

Excellent fits are obtained using the 3TM for all 32 relaxation rate profiles obtained from murine tissue covering tumour fractions in the range 0%–83%. All six fit parameters listed in Table 1 are physically meaningful and all are subject to statistical analysis to assess the significance of variability with tumour fraction.

The two fit parameters found to exhibit significant gradients *and* changes accounted for by changes in tumour fraction are the surface-to-volume ratio *x* and the bulk water dynamical time constant $\tau _b$. Both *x* and $\tau _b$ act as biomarkers. These results are consistent with the ingress of extracellular water to the intracellular volume for tumour cells compared to healthy cells as illustrated in Fig. 7.

The time constants $\tau _\ell$ and $\tau _d$ are associated with water dynamics at solid surfaces, including water bonded to proteins and other large molecules. The numerical values of both time constants are in the range 0.03–0.2 $\mu$s for all samples and these values are consistent with time constants seen in other organic material.^29^ The statistical analysis summarised in Table 2 shows that both $\tau _\ell$ and $\tau _d$ yield significant negative gradients. These results suggest that changes in the structure of the surface of tumour cell membranes, which is the largest solid component of a cell, may play a role in shaping the NMRD profiles and link to changes in water ingress and egress.

In the original analysis of their murine tissue data, Ruggiero and co-workers^19^ introduced a two-sites exchange model (2SX) to construct NMRD profiles from intracellular and extracellular contributions characterized by volume fractions and two dynamical time constants. A “slow-exchanging” component was associated with intracellular water and the “fast-exchanging” component with extracellular water. There are parallels with their 2SX model and the 3TM which also considers water in two different environments, namely bound water and bulk water. One might consider these two water environments as “two sites” with the bound water as the “solid” component justified because the bound water is the single layer of water bound to all “solid” surfaces including macromolecules. The single time constant $\tau _d$ is the timescale for water exchange between the bound water and the bulk water and $\tau _\ell$ characterises the dynamics of spins within the bound layer. Both $\tau _d$ and $\tau _\ell$ are long compared to $\tau _b$ which characterises the dynamics of the bulk water without distinguishing between intracellular and extracellular water.

Figure 2 demonstrates that the HH model alone leads to unsatisfactory fits. Satisfactory fits using the 3TM can only be achieved with the inclusion of the HM model indicating that the dynamics of water relative to rare paramagnetic ions plays an important role in describing the NMRD profiles. The similarity between the paramagnetic ion density $N_\sigma$ obtained from the 3TM fitting and typical human $\text {Fe}^{3+}$ ion density presented in Fig. 6 is excellent considering that the 3TM model was originally parametrised for hard solids rather than biological tissue^31,32^, the murine tissue samples are unlikely to be typical of the whole mouse, and not all iron in a mammal is of the form $\text {Fe}^{3+}$.

The 3TM fits to $R_1(f)$ at the lowest field $f=0.01$ MHz show that both HH and HM models contribute. See Fig. 2. The largest single contribution (56%) is from the HH model component and due to interactions between pairs of proton spins in the bound layer. This relaxation rate contribution is labelled $R_1^{\ell \ell }$ in Eq. (2) and is proportional to the surface spin density, $N_\ell$. The best fit values of $N_\ell$ lie in the range 20-35 $\text {spins/nm}^3$ which is approximately one third to one half the density of bulk water. These values are typical of organic material.^29^

The lowest field relaxation rate, $R_1(f\,=\,0.01$ MHz), is also found to be a biomarker. The difference in $R_1(f\,=\,0.01$ MHz) between healthy and tumorous tissue is largely due to changes in bound water properties captured by $R_1^{\ell \ell }$ and consistent with changes in the surface properties of tumour cells characterised by changes of $\tau _d$ and $\tau _\ell$.

The results presented here reinforce the prospects of low field $R_1$ for the identification of tumour regions in tissue. The question arises as to whether FFC NMR measurements interpreted by the 3TM could determine tumour fraction from a biopsy sample. The statistical analysis identifying two biomarkers presented here would suggest so. However, variability in the low field $R_1(f)$ between different individuals and between tumour type is not known. A clinical trial running alongside conventional assessment would answer these questions and also provide an estimate of the uncertainty in tumour fraction. Moreover, an FFC NMR profile could be generated quickly because only the lowest frequencies ($\lesssim$1 MHz) need be explored. Finally, FFC NMR may be useful in identifying diseases in biofluid such as blood which cannot be assessed using MRI.

## Additional files

Additional files include the Matlab software and its input files used to conduct the fitting, and a summary spreadsheet containing all the experimental input datasets, fitted output datasets, summary and plots.

## Data Availability

The datasets used for the current study are available as additional files. A summary spreadsheet and the Matlab files used for the fitting are also available
